# Viral emergence in marine mammals in the North Pacific may be linked to Arctic sea ice reduction

**DOI:** 10.1038/s41598-019-51699-4

**Published:** 2019-11-07

**Authors:** E. VanWormer, J. A. K. Mazet, A. Hall, V. A. Gill, P. L. Boveng, J. M. London, T. Gelatt, B. S. Fadely, M. E. Lander, J. Sterling, V. N. Burkanov, R. R. Ream, P. M. Brock, L. D. Rea, B. R. Smith, A. Jeffers, M. Henstock, M. J. Rehberg, K. A. Burek-Huntington, S. L. Cosby, J. A. Hammond, T. Goldstein

**Affiliations:** 1 Wildlife Health Center, One Health Institute, 1089 Veterinary Drive, School of Veterinary Medicine, University of California, Davis, CA 95616 USA; 2 School of Veterinary Medicine and Biomedical Sciences, School of Natural Resources, University of Nebraska, Lincoln, NE 68583 USA; 3 Sea Mammal Research Unit, Gatty Marine Laboratory, University of St Andrews, St Andrews, Fife KY16 8LB UK; 4 U.S. Fish and Wildlife Service, Marine Mammals Management, 1011 East Tudor Road, Anchorage, AK 99503 USA; 5 NOAA Fisheries, 222W. 7th Ave, Rm 552, Anchorage, AK 99513 USA; 6 Alaska Fisheries Science Center Marine Mammal Laboratory, NMFS, 7600 Sand Point Way N.E., Seattle, Washington, 98115 USA; 7 Institute of Biodiversity, Animal Health and Comparative Medicine, University of Glasgow, Glasgow, G61 1QH UK; 8 Steller Sea Lion Research Program, Alaska Department of Fish and Game, Division of Wildlife Conservation, 1300 College Road, Fairbanks, AK 99701 USA; 9 Institute of Northern Engineering, 1764 Tanana Loop, ELIF Suite 240, University of Alaska Fairbanks, Fairbanks, Alaska 99775 USA; 10 Centre for Experimental Medicine, School of Medicine, Dentistry and Biomedical Sciences, Medical Biology Centre, Queen’s University Belfast, 97 Lisburn Road, Belfast, BT9 7BL UK; 11 The Pirbright Institute, Ash Road, Pirbright, Woking, GU24 0NF UK; 12 Alaska Veterinary Pathology Service, 23834 The Clearing Dr., Eagle River, AK 99577 USA

**Keywords:** Ecological epidemiology, Pathogens, Ecological epidemiology

## Abstract

Climate change-driven alterations in Arctic environments can influence habitat availability, species distributions and interactions, and the breeding, foraging, and health of marine mammals. Phocine distemper virus (PDV), which has caused extensive mortality in Atlantic seals, was confirmed in sea otters in the North Pacific Ocean in 2004, raising the question of whether reductions in sea ice could increase contact between Arctic and sub-Arctic marine mammals and lead to viral transmission across the Arctic Ocean. Using data on PDV exposure and infection and animal movement in sympatric seal, sea lion, and sea otter species sampled in the North Pacific Ocean from 2001–2016, we investigated the timing of PDV introduction, risk factors associated with PDV emergence, and patterns of transmission following introduction. We identified widespread exposure to and infection with PDV across the North Pacific Ocean beginning in 2003 with a second peak of PDV exposure and infection in 2009; viral transmission across sympatric marine mammal species; and association of PDV exposure and infection with reductions in Arctic sea ice extent. Peaks of PDV exposure and infection following 2003 may reflect additional viral introductions among the diverse marine mammals in the North Pacific Ocean linked to change in Arctic sea ice extent.

## Introduction

Climate change and natural variability are rapidly reshaping Arctic environments^1,2^, where circumpolar declines in sea ice and rising water and air temperatures have the potential to affect diverse species of marine wildlife^3,4^. Reduction in sea ice extent and thickness impacts habitat availability, species distributions and interactions, as well as the breeding and foraging ecology of Arctic marine mammals^3,5,6^. Arctic climate change may also play an important role in marine mammal health^7^. In addition to influencing animal nutrition and physiological stress, environmental shifts may drive exposure to new pathogens in Arctic marine mammals^8^. By altering animal behavior and removing physical barriers^3^, loss of sea ice may create new pathways for animal movement and introduction of infectious diseases into the Arctic^8,9^. Although the remoteness of Arctic marine mammals creates challenges for monitoring their health, pathogen surveillance in the North Atlantic and North Pacific Oceans provides clues to polar and circumpolar infectious disease dynamics. Phocine distemper virus (PDV), a pathogen responsible for extensive mortality in European harbour seals (*Phoca vitulina vitulina*) in the North Atlantic, was identified in northern sea otters (*Enhydra lutris kenyoni*) in Alaska^8^. This finding raised the possibility that increased contact between Arctic and sub-Arctic marine mammals could result from climate change-associated reductions in Arctic sea ice extent which could alter animal movement allowing for disease transmission across the Arctic Ocean.

Phocine distemper virus was recognized as an important pathogen of phocid seals in 1988 when an outbreak caused mass mortality among European harbour seals in the North Atlantic Ocean. An early hypothesis suggested that harp seals (*Pagophilus groenlandicus*) from the Arctic may have been the source and reservoir of infection for other seal species in the Atlantic, and sympatric grey seals (*Halichoerus grypus*) may provide the link for viral transmission from Arctic species to harbour seals^10,11^. A second PDV epidemic occurred among European harbour seals from May to November in 2002, leading researchers to question whether the virus had persisted in the region in marine or terrestrial hosts, or if it was reintroduced^12^. Differences between the 1988 and 2002 PDV isolates identified through phylogenetic analysis supported introduction of a new virus. However, a PDV isolate from a harbour seal infected during a 2006 outbreak on the US Atlantic coast showed more similarity (99.3% across the genome, Genbank accession numbers NC_028249, KY229928) to the 1988 isolate, suggesting the possibility of multiple viral lineages circulating in Arctic and Atlantic seal species^13^. While Atlantic harbour seals were highly susceptible to these three strains of PDV and suffered large population losses associated with infection^14^, sympatric species, including grey seals and Arctic species such as harp and hooded (*Cystophora cristata*) seals, appear to have varying susceptibility. Although sporadic deaths do occur in these species, they are not on the order of magnitude seen with harbour seals (as reviewed in^9^).

In contrast to the North Atlantic, outbreaks of PDV have not been observed in the North Pacific Ocean. Although a small number of seropositive Kuril harbour seals (*Phoca vitulina stejnegeri*), Steller sea lions (*Eumetopias jubatus*), and spotted seals (*Phoca largha*) were reported in 1994–1999 near Japan^15^, PDV was not molecularly confirmed in these animals and seropositive results may reflect exposure to the closely related canine distemper virus (CDV). Ringed seals (*Phoca hispida*) that were seropositive for PDV were detected along Canada’s Northwest Territories near northern Alaska in 1993 and 1994^10^, but serologic surveys of Pacific harbour seals (*Phoca vitulina richardsii*), Steller sea lions, and northern sea otters off Alaska prior to 2000 showed little evidence of exposure to distemper viruses, and PDV had not been identified as a cause of illness or death^16–18^. PDV was not confirmed in the North Pacific Ocean until virus was detected in northern sea otters sampled in 2004, which raised a number of questions regarding the timeline of introduction into the North Pacific, how the virus reached sea otters, and what role the virus may play in North Pacific marine mammal illness and mortality.

Nomadic Arctic seals with circumpolar distributions (e.g. ringed and bearded, *Erignathus barbatus*, seals) and geographic ranges that intersect with those of harp seals, may be carriers of PDV to the North Pacific. Overlapping distributions of marine mammal species may then allow for transmission of the virus to other ice seal species (spotted seals and ribbon seals, *Histriophoca fasciata*) and sub-Arctic species such as Steller sea lions, northern fur seals (*Callorhinus ursinus*), and northern sea otters. We hypothesized that reduction in Arctic Ocean sea ice increased contact among Arctic seal species, leading to PDV introduction into the North Pacific Ocean and infection in sub-Arctic species. We evaluated exposure to and infection with PDV in sympatric ice-associated seals, northern fur seals, Steller sea lions, and northern sea otters sampled in the North Pacific Ocean from 2001–2016 to explore the timing of PDV introduction, patterns of transmission following introduction, and environmental and other risk factors associated with the emergence of PDV across the North Pacific Ocean. We incorporated satellite telemetry data from ongoing ecological studies of seals and Steller sea lions, which provided a unique opportunity to combine animal movement and epidemiologic data to understand the potential spread of PDV.

## Methods

### Marine mammal capture and sampling

Ice-associated seals (bearded seals, ribbon seals, spotted seals, and ringed seals), northern fur seals, Steller sea lions, and northern sea otters were live-captured and manually restrained, sedated, or anesthetized using established methods during ongoing field studies^19–24^. From 2001–2016, paired blood and nasal swab samples were collected when possible^25,26^. Blood and tissues were also collected from dead animals harvested for subsistence^27^ or stranded and recovered on beaches.

### Ethics statement

All animal sampling and testing methods were carried out in accordance with relevant guidelines and regulations. Animals were sampled under experimental protocols approved by the US Fish and Wildlife Service’s Marine Mammals Management Office (Permit MA041309-1 for northern sea otters) and the Alaska Fisheries Science Center’s Marine Mammal Laboratory (Permits 14327-01 for northern fur seals, 782-1532 for Steller sea lions, and 782-176 and 782-1676 for ice-associated seals). Experimental protocols for Steller sea lion samples collected by Alaska Department of Fish and Game (ADF&G) were authorized under MMPA permits 358-1564, 358-1769, 358-1888, 14325, and 18537 and under ADF&G IACUC protocols 03-0002, 06-07, 09-28, 2011-025 and 2013-30. Samples were tested under the Biological Use Authorization BUA# R2407 approved by the University of California Davis Institutional Biological Committee.

### Serologic and molecular analysis

Serology to detect antibodies to PDV (n = 1,227 animals tested 2001–2013) was performed by micro neutralisation^28^. Briefly, 1 in 10 dilutions of serum were made followed by 2-fold dilutions in high glucose DMEM medium (Gibco, USA). Cell monolayers were examined for cytopathic effect and end point neutralisation titres determined by the Reed and Muench method. Sera were tested against PDV/USA2006 and PDV/DK/2002 strains. As the earliest molecularly confirmed PDV infection in the North Pacific was detected in 2004^8^, a subset of sera from Steller sea lions (n = 80) sampled 2001–2004 in Russia, the Aleutian Islands, the Gulf of Alaska, and Southeast Alaska was tested by micro neutralization against the related morbillivirus, canine distemper virus (CDV/Snyder Hill/wild type strain).

Serologic titres were log transformed and cumulative percent was plotted against the log(10) titre to determine the appropriate cut-off to classify positive animals for further analyses (i.e. the geometric mean). The cut-off for PDV seropositive animals used was ≥1:32 (log10 = 1.5, Supplementary Fig. 1). This threshold level was used to minimize the likelihood of false positives, providing a robust estimate of seropositivity. Steller sea lions tested for both PDV and CDV were classified as seropositive for one virus based on the highest titre measured in each sample.

For molecular analysis, total RNA was extracted using Tri Reagent™ (Sigma) and complementary DNA transcribed (Superscript III, Invitrogen) with random nonamers from nasal swabs, blood, and tissues from animals sampled 2001–2002 and 2004–2016 (n = 1,994). Samples were screened for a fragment of the morbillivirus phosphoprotein (P) gene using a Real-Time Quantitative PCR designed for the study (Forward primer (RT‐P2 s): 5′‐CAT GCT AAT GGA GGA AGA GTT GAC T‐3′; Reverse Primer (RT‐P2 as): 5′‐GTT CTC CCA TCC CTC TTT TGG‐3′; Probe (PDV P2): 5′d FAM‐CTC TGC TTG GCA CAG GCC ACA ATG‐BHQ‐1 3′) to quantify viral load or a heminested PCR with universal morbillivirus primers followed by PDV and CDV-specific primers to obtain products for sequencing^29^. Heminested PCR products were visualized by agarose gel electrophoresis and products of the expected size were cloned before sequencing. Sequences were edited manually in Geneious Pro v9.1.3 (Biomatters Ltd., Auckland, NZ) and compared with known sequences in the Genbank database. Viral nucleotide sequences were aligned with ClustalW and MUSCLE, and phylogenetic trees constructed comparing the corresponding P-gene fragments (389 bp) of known morbilliviruses using Bayesian (Mr. Bayes) algorithms v3.2.6^30^. A subset of duplicate samples (n = 60 northern fur seal nasal swabs) was submitted to The Pirbright Institute, Non-Vesicular Disease Reference Laboratory in the UK for confirmatory testing. Nasal swab, blood, or tissue samples with a C_t_ < 37 and/or confirmed by sequencing were considered positive.

### Assessing Arctic Ocean sea ice extent and open water routes from the north Atlantic to north Pacific Oceans

**Figure 1:**
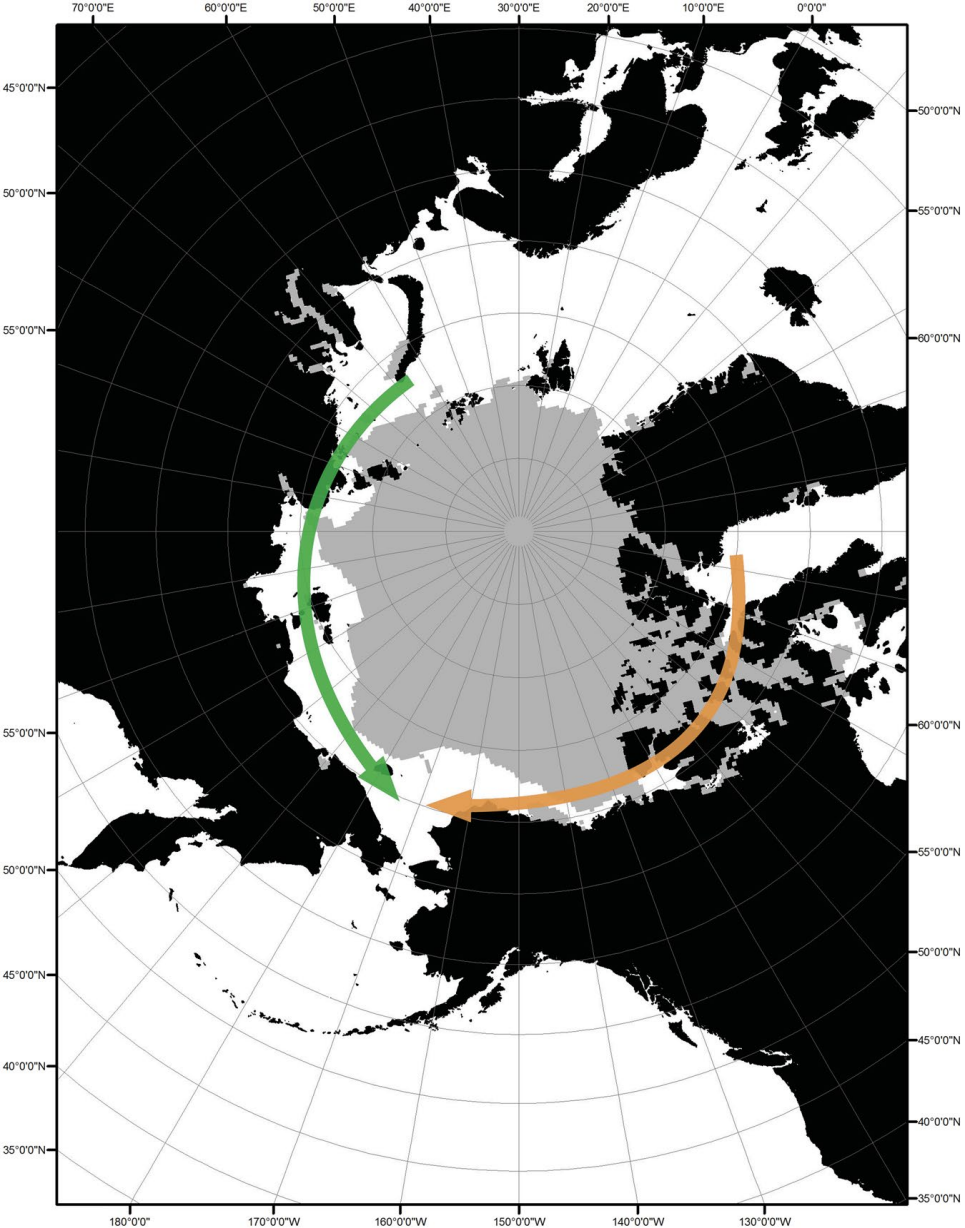
Potential routes for movement of seals infected with PDV through the Arctic Ocean opened by reductions in sea ice extent. Routes along northern Russia (green) and northern Canada (orange) are shown with August 2002 sea ice extent. Changes in historic sea ice barriers may facilitate Arctic and sub-Arctic seal movement and contact that was not possible in years prior to PDV detection, allowing for introduction of PDV into the Northern Pacific Ocean.

Sea ice in the Arctic Ocean reaches its minimum annual extent, which varies in size (area covered) and shape (geographic extent), during August to September. In some years, open water routes are created along its edges connecting the northern Atlantic and Pacific Oceans. Additionally, a trend in reduced minimum extent of the Arctic sea ice has been linked to long-term climate change^2^. Two potential routes of contact near the sea ice edge exist across the Arctic Ocean between the Atlantic and Pacific – (1) along the northern Russian coast from the Barents Sea and eastern North Atlantic and (2) along the coast and islands of northern Canada from the western North Atlantic (Fig. 1). Contact with potential PDV reservoir species is possible on either route, as harp seals breed in the Barents Sea, the coast of Greenland, and the eastern coast of Canada^31^. To evaluate associations between climate change-related reductions in Arctic sea ice extent and PDV exposure or infection, we assessed presence of Russian and Canadian open water routes through the Arctic Ocean in August-September of the year prior to animal sampling (e.g., ice extent in August-September 2002 was used for animals tested for PDV in 2003). Presence of open water routes in a given year (2000–2015) was manually assessed in ArcGIS v10.3.1 (ESRI, Redlands, California, USA) using satellite imagery of waters bordering northern Russia (from the Barents Sea to the Chukchi Sea) and northern Canada (from the eastern Canadian Arctic to the Canadian Northwest Territories) from publicly available, monthly Arctic sea ice extent shapefiles archived 1979-present by the National Snow and Ice Data Center^32^. These shapefiles classify areas of the Arctic Ocean with sea ice concentration less than 15% as open water.

### Phocine distemper virus prevalence and risk factor analyses

PDV seroprevalence and prevalence of viral infection (PCR detection of viral nucleic acid) with 95% exact confidence intervals were calculated for each species and all species combined by year of sample collection. Observed prevalence was calculated as the number of positive (seropositive or PCR positive) animals divided by the total number of animals tested. To evaluate infection annually, we limited calculations for prevalence of viral infection to animals actively shedding PDV (PCR detection of PDV in nasal swabs from live captured and subsistence harvested animals). Prevalence calculations and statistical analyses described below were performed using R v3.3.2^33^ with a significance level of α = 0.05.

Associations between PDV exposure (seropositive titre) or viral infection (PCR positive) and demographic variables (animal group (ice-associated seals, Steller sea lions, northern fur seals, and northern sea otters), age class, and sex), disposition (live captured or subsistence harvested vs. found dead), and presence of an open water route through Arctic Ocean sea ice were assessed in mixed effects logistic regression models using the lme4 package^34^. We evaluated three open water route variables: (1) presence of an open water route along the Russian coast, (2) presence of an open water route along the Russian coast in a year following closed sea ice, and (3) presence of an open water route along the Canadian coast. Sampling region (Bering Sea, Pribilof Islands, Southeast Alaska, Eastern Gulf of Alaska, Central Gulf of Alaska, Western Gulf of Alaska, Eastern Aleutian Islands, Central Aleutian Islands, Western Aleutian Islands, or Russia) was determined based on individual animal sampling locations and included as the random effect in mixed effects models. Risk factors associated with PDV exposure or viral infection in univariable regression models (P ≤ 0.20) were incorporated into multivariable models. Following inclusion of statistically (P ≤ 0.05) and biologically significant variables, multivariable models were compared using Akaike’s Information Criterion (AIC) to select the most parsimonious model. Adjusted odds ratios with respective 95% confidence intervals were calculated for levels of each risk factor.

### Satellite tag data and spatial analyses

Telemetry databases maintained by the Alaska Fisheries Science Center’s Marine Mammal Laboratory and ADF&G were queried retrospectively for bearded seals, northern fur seals, and Steller sea lions tagged at times and locations that coincided with the geographic area and, when possible, years of PDV sample collection. Location data received from Service Argos were filtered^35^ using a swim speed of 2 m/s for bearded seals (n = 7), northern fur seals (n = 136), and Steller sea lions (n = 77). Filtered data were processed with a continuous-time correlated random walk (CTCRW) model to predict animal locations for time intervals (e.g. 20 min or 1 hour) corresponding to the timing of conductivity readings (i.e. wet or dry) collected for each species^36,37^. This model provided swim speed estimates (m/s) between sequential predicted locations, which were used to obtain the average median swim speed (m/s) for each species at sea.

The duration of viral shedding for PDV in pinniped species is unknown, but previous estimates of the combined latent (post-exposure, but not shedding virus) and infectious (shedding virus) periods in European outbreaks range from 11–18 days in harbour seal outbreak models^38^. Mean latent periods of 6–10 days and mean infectious periods of 9–13 days were used to model CDV transmission among wild canids in terrestrial systems^39^. A modeling study of an outbreak of another marine morbillivirus, Dolphin morbillivirus, estimated a mean infectious period of 8 days, with an upper bound of 24 days^40^. To capture variability in marine mammal latent and infectious periods and to assess the geographic distances that an animal infected with PDV could move and potentially transmit the virus (i.e. movement during latent and infectious periods), the distance moved by a PDV-infected individual of a given species was estimated for 1, 2, and 4 week periods (calculated as the median speed (m/s) of movement multiplied by the number of hours in the time period). The resulting estimated 1, 2, and 4 week movement distances in kilometers (km) were used to spatially buffer known animal sampling locations in ArcGIS v10.3.1 to map the distance the virus could be transported via animal movement. A subset of animals with PDV serologic results (seropositive and seronegative animals) and/or PCR testing results (only PCR negative animals) had telemetry data available. For seropositive animals, 1, 2, and 4 week post-sample collection movement tracks were overlaid on the spatial distance buffers to compare observed and expected movement of the virus with host movement.

## Results

Samples were collected from 2,530 live and 165 dead ice-associated seals (bearded, ringed, ribbon, and spotted seals), Steller sea lions, northern fur seals, and northern sea otters in the North Pacific Ocean between 2001 and 2016; not all species were sampled in all years (Supplementary Table 1). Sampling locations ranged from Southeast Alaska to Russia along the Aleutian Islands as well as the Bering, Chukchi, and Beaufort Seas.

**Figure 2:**
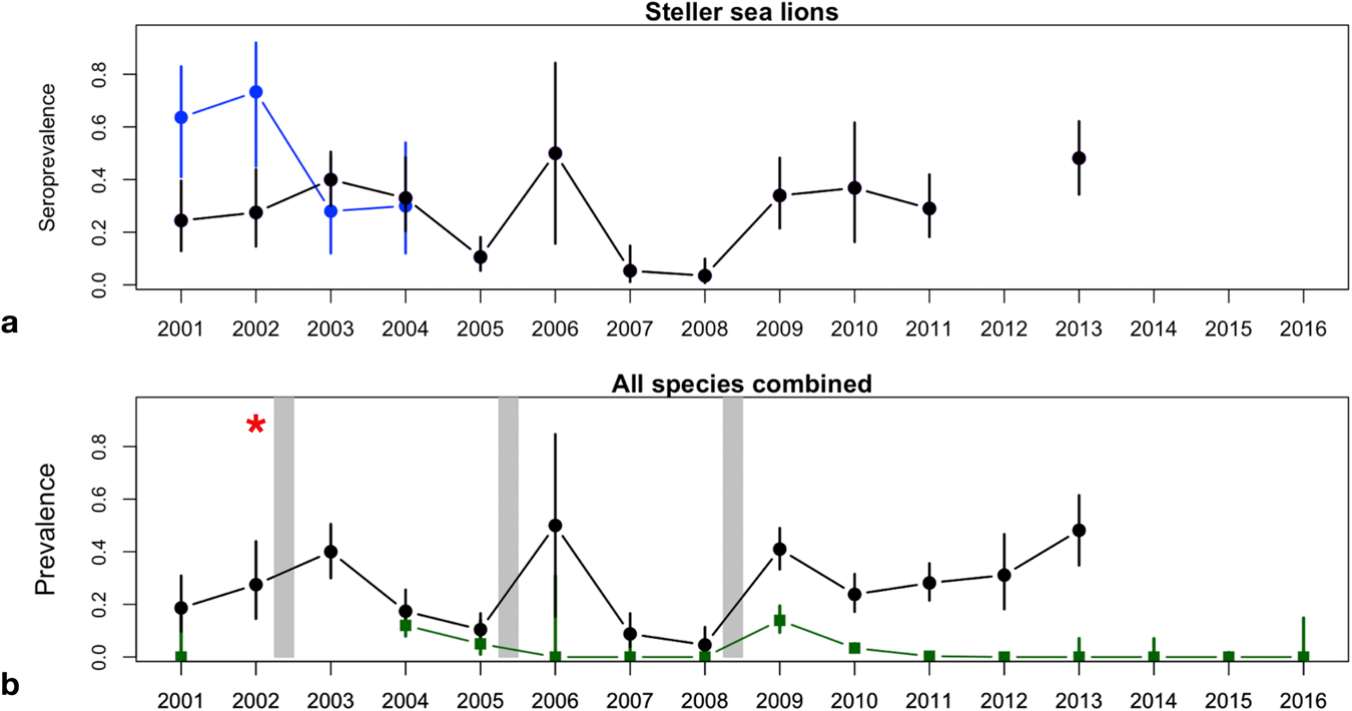
(**a**) Yearly seroprevalence for antibodies to PDV in Steller sea lion pups, juveniles, and subadults (black dots) with seroprevalence of canine distemper virus antibodies (blue dots) measured in a subset of Steller sea lions (n = 80); (**b**) PDV seroprevalence (black dots) and viral infection prevalence (PDV nucleic acid detected from nasal swabs; green squares) for all species combined (ice-associated seals, Steller sea lions, northern fur seals, and northern sea otters) from 2001–2016. Error bars represent 95% exact confidence intervals (CI). A 95% CI was not included for viral infection prevalence in 2008 as only one animal was tested. Presence of an open water route through Arctic sea ice along the northern Russian coast following a year of closed sea ice (grey bars) was significantly associated with animals testing seropositive or PCR positive for PDV. The strain of PDV responsible for an outbreak in harbour seals in the North Atlantic Ocean during 2002 (red star) was detected in PCR positive animals in the North Pacific Ocean throughout the study period.

Steller sea lion samples for serology and/or PCR testing were available for the majority of the study period (13 of 16 years) and suggest peaks in PDV exposure and infection beginning in 2003. Animals sampled earlier in the study period showed greater evidence of exposure to CDV. Comparisons of seroprevalence of PDV and CDV in a subset of 80 Steller sea lions tested for both viruses 2001–2004 suggest exposure to CDV in 2001 (64%; 14 of 22 animals tested) and 2002 (73%; 11 of 15 animals tested, Fig. 2a), shifting to exposure to PDV in 2003 (72%; 18 of 25 animals tested) and 2004 (70%; 14 of 20 animals tested).

**Figure 3:**
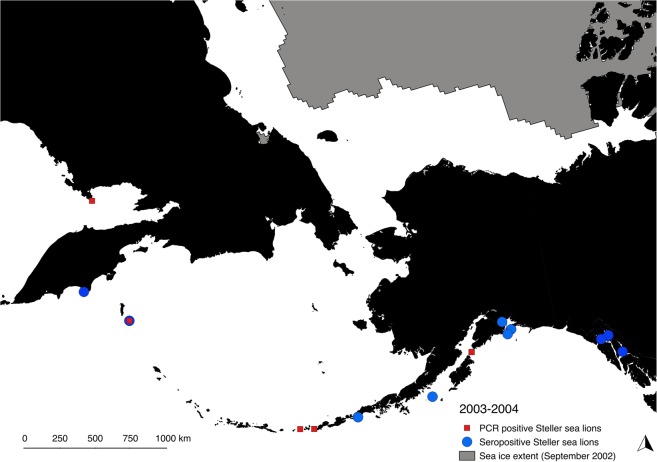
Locations of PDV seropositive and PCR positive Steller sea lions detected from Southeast Alaska to eastern Russia in 2003–2004. Sea ice is shown at its minimum extent in September 2002 prior to widespread detection of PDV in the North Pacific Ocean beginning in 2003.

Following reduced Arctic sea ice extent and presence of an open water route along Russia in August 2002, serologic and PCR results demonstrated widespread exposure to and infection with PDV in Steller sea lions in the North Pacific Ocean in 2003–2004. Seropositive sea lion pups (2–11 months of age) and juveniles were present at rookery and haul-out locations in the western Pacific/eastern Russia (Koslov Cape and Medny Island), Aleutian Islands (Ugamak Island), Gulf of Alaska (Chowiet Island, Glacier Island, Perry Island, Seal Rocks), and Southeast Alaska (Benjamin Island, Gran Point, Little Island, Southwest Brothers Island) in 2003–2004 (Fig. 3), with over 30% of animals seropositive in both years (38 positive/95 tested in 2003 and 16 positive/48 tested in 2004, Supplementary Table 2). Although no PCR samples were available for 2003, 21 Steller sea lions (12.3% of 171 tested) were PCR positive for PDV (viral RNA detected in nasal swab samples from live-captured animals) in 2004. The PCR-positive animals were 2–3 month old pups from widespread locations across the North Pacific Ocean (Fig. 3) including Russia (Medny and Yamsky Islands), the Aleutian Islands (Adugak and Yunaska Islands), and the Gulf of Alaska (Sugarloaf Island).

After the widespread PDV exposure and infection in 2003–2004, seroprevalence and the proportion of PCR positive Steller sea lions decreased in 2005 (10.6% and 5.3%). A higher seroprevalence (50%) was detected among a small sample (n = 8) of 9-month old pups captured at one location in the Gulf of Alaska in March-April of 2006. These animals were in the 2005 birth cohort, and may have been exposed to the virus in late 2005 or early 2006 following the decline of maternal antibodies. Seroprevalence decreased further in 2007 and 2008 (5.4% and 3.5%, respectively), and all Steller sea lions tested negative by PCR from 2006–2008 (n = 27). Similarly, PDV titres decreased from 2005–2008 (Supplementary Fig. 3a). However, Steller sea lion seroprevalence rose 10-fold from 2008 to 34% in 2009, remaining high in 2010, 2011, and 2013 (36.8%, 29%, and 48.1%; Fig. 2a).

Patterns of seroprevalence and viral infection (PCR) in all sampled species support the second peak of PDV exposure and infection in 2009 (Fig. 2b, Supplementary Table 2). The high seroprevalence (68.7%) observed in ice-associated seals of all ages in 2009 decreased significantly in 2010 (6.3%). Serologic titres also decreased from 2009–2010 (Supplementary Fig. 3b), and the proportion of PCR positive ice-associated seals decreased, from 16% (10 positive/62 tested) in 2009 to 0% (43 tested) in 2010. Seroprevalence and serological titres increased from 2009–2012 in northern fur seals (Supplementary Fig. 3c), whereas the proportion of PCR positives decreased from 11.6% (7 positive/60 tested, confirmed by testing a subset of samples at The Pirbright Institute, Non-Vesicular Disease Reference Laboratory) in 2009 and 4.3% (12 positive/280 tested) in 2010 to zero in 2011 and 2012 (57 and 250 animals tested).

Prevalence of PDV viral shedding for all species combined peaked in 2004 and 2009 (no PCR data were available for 2003; Fig. 2b, Supplementary Table 2). Controlling for animal group, age class, and disposition (live vs. dead), the odds of viral infection were 9.2 times higher (95% CI: 5.1–16.8) in animals sampled in 2004 and 2009 relative to other years when animals were tested by PCR (Table 1, model 2). Detection of virus in nasal swabs from live or subsistence harvested animals, suggesting active viral shedding, indicated recent infection. Similar to the decrease detected from 2004 to 2005 (Fig. 2b), viral shedding in all species fell four-fold from 2009–2010 and remained low from 2011–2016. Viral presence was also detected in tissues from dead northern sea otters in 2006 (3 positive/30 tested), 2007 (4/22 tested), 2008 (4/52 tested), and in 2009 (2/9 tested), and from dead SSL pups and fetuses in 2005 (1/8 tested) and in 2012 (2/5 tested). Detection of viral nucleic acid in tissues from dead animals demonstrates that virus is present but doesn’t necessarily indicate viral replication and shedding. Therefore, determining the timing of infection for dead animals that test PCR positive for PDV in their tissues is challenging. Sequences of the short fragment of the P-gene from PCR-positive animals of all species and across years and locations clustered together and were most similar to the PDV2002 strain isolated from European harbour seals infected during the 2002 PDV outbreak (Supplementary Fig. 2).

**Table 1:** Risk factors for PDV exposure or viral infection (Model 1) and viral infection (Model 2) in marine mammals sampled in the North Pacific Ocean 2001–2016.

Model Number: dataset	Risk Factor (reference category)	Adjusted Odds Ratio		95% Confidence Interval		P-value
1: Viral exposure or infection (serologic or PCR) status	**Open water along northern Russia following a closed ice year**					
	(No: 2000-1, 2003-4, 2006-7, 2009-15)		1	—	—	
	Yes: 2002, 2005, and 2008		3.1	(2.2–4.2)	<0.01*	
	**Open water along northern Canada**					
	(Ice present along northern Canada)		1	—	—	
	Open water along northern Canada		0.7	(0.5–0.9)	<0.01*	
	**Age**					
	(Adult)		1	—	—	
	Juveniles and Subadults		0.5	(0.3–0.7)	<0.01*	
	Fetuses, Pups, Young of Year		0.5	(0.3–0.9)	0.01	
	**Marine mammal group**					
	(Steller sea lion)		1	—	—	
	Ice-associated seals		1.6	(0.6–4.4)	0.38	
	Northern fur seals		0.5	(0.2–1.6)	0.26	
	Northern sea otters		0.5	(0.3–0.9)	0.02*	
2: Viral infection (PCR) status	**Peak year**					
	No: 2001, 2002, 2005-8, 2010-16		1	—	—	
	Yes: 2004 and 2009		9.2	(5.1–16.8)	<0.01*	
	**Age**					
	(Adult)		1	—	—	
	Juveniles and Subadults		0.4	(0.2–0.9)	0.04*	
	Fetuses, Pups, Young of Year		1.6	(0.7–3.8)	0.28	
	**Marine mammal group**					
	(Steller sea lion)		1	—	—	
	Ice-associated seals		2.2	(0.6–8.3)	0.26	
	Northern fur seals		3	(0.7–12.7)	0.14	
	Northern sea otters		1.2	(0.3–5.3)	0.79	
	**Sampling status**					
	(Healthy: live capture or subsistence harvest)		1	—	—	
	Dead stranded		6.2	(1.9–20.6)	< 0.01*	

^*^Statistically significant association with PDV exposure or infection, α = 0.05.

Increased levels of PDV exposure or infection in the sampled animals were associated with the presence of an open water route along the Russian coast. Reductions in sea ice extent created open water routes to the Pacific Ocean along the northern Russian coast (Fig. 1) in August and/or September of 2000, 2002, 2005-2006, and 2008–2015, with the open water routes in 2002, 2005, and 2008 following a year in which sea ice blocked passage through at least part of the Arctic Ocean bordering Russia’s coast. When controlling for animal group and age class, presence of an open water route along the northern Russian coast following a year in which the Arctic sea ice along the Russian coast was closed was significantly associated with PDV exposure or infection (Table 1, model 1). The odds of PDV exposure or infection were 3.1 times higher (95% CI: 2.2–4.2) in animals sampled in a year following one of these complete openings in the Russian coast sea ice in August–September of the previous year. In contrast, presence of an open water route along the Canadian coast, which existed in August and/or September of 2006–2007, 2010–2012, and 2015, was negatively associated with PDV exposure or infection (OR: 0.7; 95% CI: 0.5–0.9).

**Figure 4:**
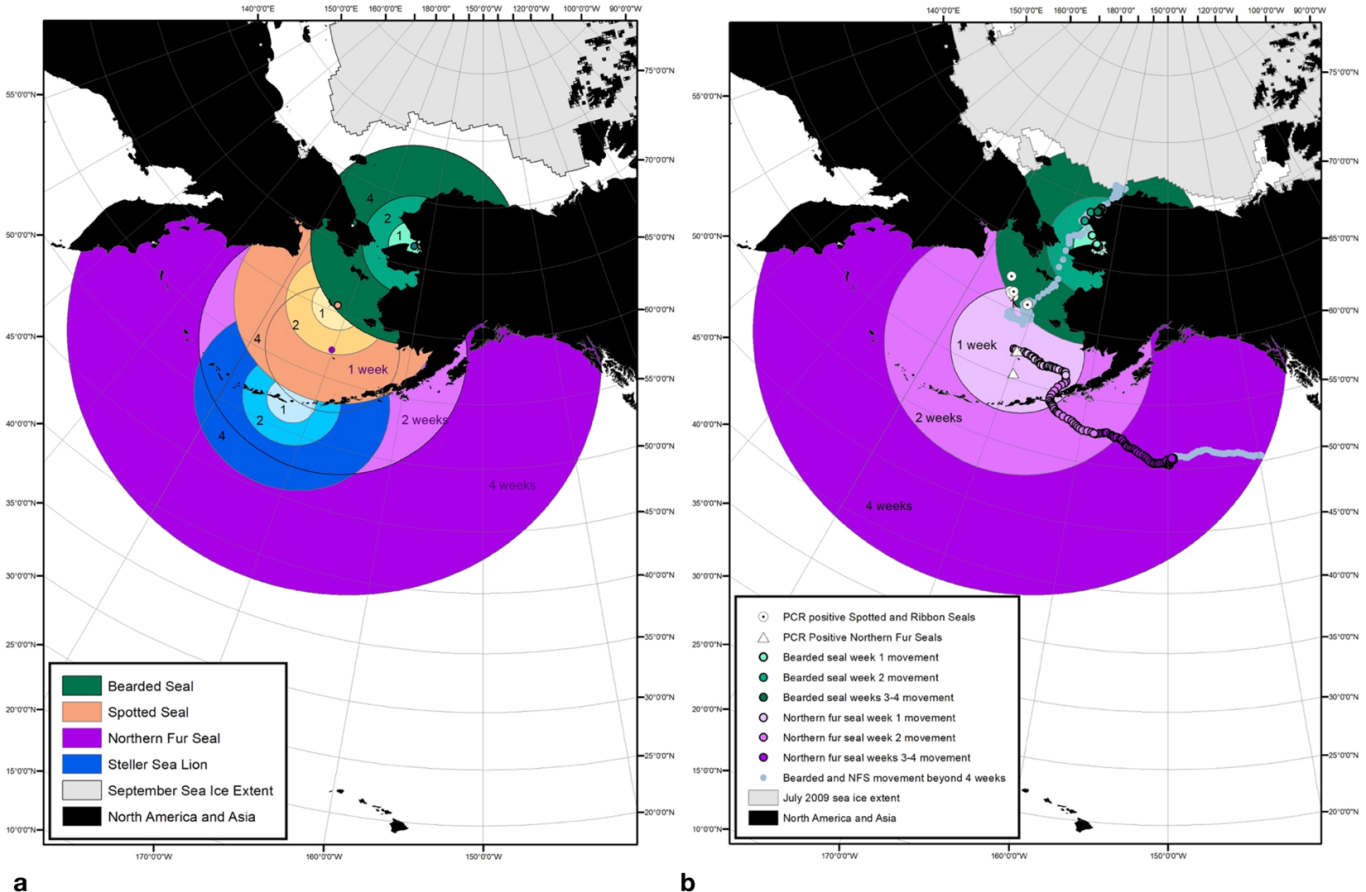
(**a**) Estimated distances animals can travel during the PDV latent and infectious period (1 week, 2 weeks, and 4 weeks) illustrating the areas where viral transmission could occur, based on median travel speeds calculated for satellite-tagged bearded seals (green circles), spotted seals (orange), Steller sea lions (blue), and northern fur seals (purple). (**b**) Recorded tracks of a PDV seropositive bearded seal followed in July 2009 and a seropositive northern fur seal followed in November 2010 shown with sympatric PCR positive spotted seals, ribbon seals, and northern fur seals sampled 2009–2010. Sea ice is shown at its minimum extent in September (panel a) and retreating the following July after reaching a maximum winter extent (panel b).

Infected pinnipeds shedding PDV have the potential to reach nearby rookeries and haul-outs as well as more distant areas inhabited by conspecifics or other species (Fig. 4a). Median speeds of travel calculated from satellite-tagged bearded seals, northern fur seals, and Steller sea lions were 0.36 m/s, 0.89 m/s, and 0.32 m/s, respectively. Bearded seal speeds were used as a conservative movement estimate for wide-ranging spotted seals. Estimated movement distances for the 1, 2, and 4-week periods were 210 km, 420 km, and 840 km for bearded and spotted seals; 535 km, 1,070 km, and 2,140 km for northern fur seals; and 194 km, 388 km, and 776 km for Steller sea lions. Recorded movement tracks from one PDV seropositive bearded seal sampled in 2009 and one seropositive northern fur seal sampled in 2010 occurred within the estimated 1, 2, and 4-week movement distances for each species (Fig. 4b). Movement tracks for three additional PDV seropositive animals (two bearded seals and one northern fur seal) sampled in 2009 also fell within their species-specific 1, 2, and 4-week estimated movement zones. PCR positive spotted seals, ribbon seals, and northern fur seals sampled 2009–2010 were detected in close proximity to bearded seal or northern fur seal movement tracks (Fig. 4b).

## Discussion

Serologic, PCR, and sequencing results provide evidence for (1) widespread exposure to and infection with PDV across the North Pacific Ocean beginning in 2003; (2) viral transmission across multiple marine mammal species; and (3) decline of viral infections following peaks of exposure in 2003 and 2009, with sporadic detection of PCR positive animals from 2005–2008 and 2011–2016. Peaks of PDV exposure and infection followed reductions in Arctic sea ice extent, potentially linking climate change and interannual variability with introduction of PDV into the North Pacific Ocean.

During August and September of 2002, sea ice extent in the Arctic Ocean reached a new minimum^32^. Open water along most of northern Russia linked the North Pacific Ocean to the eastern North Atlantic Ocean (Fig. 1), where European harbour seals were dying in an epidemic of PDV that began in May 2002. Reduction in the sea ice that previously created a barrier to animal movement across the Arctic Ocean may have allowed for increased contact between harp seals, the proposed Arctic reservoir of PDV^10,11,41^, and circumpolar seal species (including bearded seals and ringed seals). As the strain of PDV detected in North Pacific marine mammals is most similar to the strain isolated during the 2002 outbreak in European harbour seals, seals may have moved it from the North Atlantic outbreak or they may share a common source. If the 2002 PDV strain was circulating in harp seals that breed in the Barents Sea or near the coast of Greenland, it could have been carried south to grey seals and/or sympatric European harbour seals in early 2002 and across the Arctic Ocean to the North Pacific Ocean in August-September. Evidence of widespread PDV exposure in the North Pacific was first detected in Steller sea lions in our study in 2003, with 20 two month-old pups, 10 four to five month-old pups, and 8 animals 16 months of age or older testing seropositive. Two month-old pups likely have maternal antibodies reflecting their mothers’ prior exposure^42^, which creates a challenge for identifying the timing of exposure. However, the seropositive four and five month-old pups identified in 2003 suggest that sea lions in the 2003 birth cohort were exposed to circulating PDV.

Sporadic reports suggest morbillivirus exposure may have occurred prior to 2003 in Japan^15^ and Alaska^18^ (2 of 160 harbour seals in Alaska tested positive for PDV and were identified as likely false positives). Seropositive Stellar sea lions detected in our study in 2001 and 2002 suggest likely exposure to CDV not PDV as titres to CDV were highest in those years. While it is possible that PDV infection occurred in some sites in the North Pacific Ocean prior to 2003, exposure and molecularly-confirmed infection in Steller sea lion pups sampled from Southeast Alaska to Russia in 2003–2004 (Fig. 3) and PDV seropositive Kuril harbour seals near Japan in 2004^15^ suggest that widespread PDV infection in the North Pacific began after 2002 and that the virus rapidly reached geographically distant populations across the North Pacific Ocean after introduction.

Northern sea otters, with range overlap with Steller sea lions, tested positive by serology and PCR beginning in 2004, providing early evidence for cross-species transmission in the North Pacific Ocean. Detection of PDV exposure and infection in northern fur seals and four species of ice-associated seals during the second peak of infection in 2009 suggests additional cross-species transmission occurred. In general, morbilliviruses are transmitted through respiratory droplets or by contact such as through fluids^43^ when animals are in close proximity at locations like rookeries. While some pinniped species in Alaska use separate rookeries or haul-outs, cross-species mixing has been observed at ice and land-based locations. Cross-species PDV transmission may also be possible among animals feeding and/or surfacing in close proximity in open water. Telemetry studies and field observations have demonstrated likely overlap in foraging areas such as the prey-rich waters along the Bering Shelf (J. London, pers. obs.).

Given the limited serologic data for species other than Steller sea lions and the sparse PCR data for each species across the study period, it is difficult to determine if PDV was reintroduced in association with changing sea ice extent following the 2003–2004 peak of exposure and infection in the North Pacific Ocean, or whether viral transmission was maintained among marine mammal species. While we may not have detected PDV infection in certain years with smaller sample sizes (Supplementary Table 2), the combined serologic and PCR data provide support for a second peak of infection in 2009. The short sequence fragments of PDV amplified in infected North Pacific marine mammals throughout our study are similar to each other and to the 2002 UK outbreak strain. Strains from the 1988 European outbreak and the 2006 outbreak along eastern North America differ from the 2002 European outbreak and the North Pacific sequences, suggesting that multiple viral strains may be circulating in Arctic reservoir hosts. Sequences most similar to the 2002 PDV isolate in North Pacific species could be due to reintroduction from Arctic hosts or endemic transmission within the North Pacific. In the North Atlantic Ocean, outbreaks of PDV in European harbour seals appear to sweep through the population within a single year and then fade out until the virus is reintroduced likely through cross-species contact with grey seals or harp seals^41^. In combination with the proportion of susceptible marine mammals in the population at a given point in time, viral reintroduction via contact between Arctic reservoir species may also shape cycles of exposure and infection in the North Pacific Ocean. As seropositive ringed seals in the Northwest Territories of Canada were reported in the 1990’s^10^, Canadian circumpolar seals could potentially serve as a source of PDV to North Pacific marine mammal species. However, sea ice extent and genetic data from our study support introduction along the Russian route. Openings in the sea ice along the Russian coast were linked to the increased likelihood of PDV exposure or infection (Table 1), and peaks of viral exposure in 2003 and 2009 occurred after a year with low sea ice extent and open water along the Russian coast, suggesting that multiple introductions may have occurred.

Alternatively, cross-species transmission resulting in PDV circulation among ice-associated seals, Steller sea lions, northern fur seals, and northern sea otters might also result in peaks of infection following introduction. The estimated number of Steller sea lions, northern fur seals, and ice-associated seals in Alaskan stocks^44^ exceeds the critical community size estimated to be necessary to sustain endemic PDV transmission in European harbour seals (~300,000 animals)^38^. The probability of long-term pathogen persistence is likely to increase in systems with multiple hosts capable of cross-species disease transmission^45^. Differences in the density and spatial distribution of a single species may also enhance the potential for disease persistence. Although species like Steller sea lions and northern sea otters are distributed across the North Pacific, the patchy distribution of haul outs and rookeries could prolong the duration of viral transmission among species following PDV introduction^38^. Low density, patchy groups of terrestrial carnivores spread across a large landscape can maintain transmission of CDV^39^. Population age and sex structure, seasonal patterns of movement and intra-and inter-specific contact, and variation in host susceptibility to viral infection and disease severity could also contribute to the potential for endemic viral transmission. Spatially explicit epidemiologic modeling integrating movement data from satellite-tagged marine mammals with seroprevalence and viral infection prevalence trends may help to elucidate whether circulation among species or reintroduction associated with ice change is driving viral transmission in the North Pacific.

Linking movement data from satellite-tagged marine mammals with biological information on viral shedding illustrates that exposed animals have the potential to carry PDV long distances. Movement of PDV seropositive bearded seals and northern fur seals occurred within their species-specific predicted viral transmission distances and in close proximity to known locations of PCR positive individuals from all species tested. These data demonstrate the potential for animals exposed to PDV to carry the virus to areas with conspecifics and sympatric species. Data from satellite-tagged Steller sea lions suggest infectious animals could move over 100 km in one week, reaching nearby and distant rookeries (Fig. 4a). Bearded seals and northern fur seals have the potential to move over 200 km and 500 km in one week, respectively. Spotted seals and ribbon seals may bridge the gap between northern ice-associated seals and Steller sea lions and northern fur seals living in the southern Bering Sea (Fig. 4a). The ability to move long distances and timing of movements associated with life history cycles likely influence transmission patterns in the North Pacific Ocean and the potential to transmit PDV to species living in southern habitats.

Finally, the animal health impacts of PDV in North Pacific species are unknown, but may be quite different from the outbreaks in the Atlantic Ocean where the virus has caused extensive mortality in harbour seals^46^. Although no mortality events have been documented with PDV infection in Pacific species, the virus may have contributed to sporadic northern sea otter deaths, including those that occurred during an unusual mortality event from 2004–2006^8^, as well as to sporadic Steller sea lion deaths (PCR positive tissues in dead animals in this study). We found evidence of PDV viral infection in apparently healthy Steller sea lions, northern fur seals, and ice-associated seals sampled live or through subsistence harvest throughout the study. As with grey seals and Arctic seal species in the North Atlantic, PDV may be able to persist in Arctic and some sub-Arctic species in the North Pacific without causing widespread disease. The current impacts of PDV associated disease and deaths in marine mammal species in the North Pacific Ocean are unknown, but the potential for outbreaks in sensitive species highlights the importance of understanding spatio-temporal PDV transmission in this environment. Climate change-driven reductions in sea ice extent in the Arctic Ocean are projected to increase^47^ and open water routes along the northern Russian coast have occurred every August and/or September since 2008. The health impacts of this new normal in the Arctic are unknown, but association of open water routes through Arctic sea ice with increased PDV exposure or infection suggest that opportunities for PDV and other pathogens to cross between North Atlantic and North Pacific marine mammal populations may become more common.

## Data and code availability

Sequences obtained in this study were submitted to Genbank with the following accession numbers:

FW07098/Brain/Kodiak/2007_PDV (MH392513)

HF2010-2019/Nasal/Bering_sea/2010_PDV (MH392514)

NFS_G105Y/Nasal/St_George/2010_PDV (MH392515)

SSL_Y389/Nasal/Yamsky/2004_PDV (MH392516)

NFS_SAM81/Nasal/St_Paul/2012_PDV (MH392517)

PL2009-2016/WBC/Bering_sea/2009_PDV (MH392518)

SSL_2012-032/Placenta/Kodiak/2012_PDV (MH392519)

SSL_YUN04-19/Nasal/Yanuska/2004_PDV (MH392520)

Animal species, location, and PDV serology and PCR testing data are summarized in the electronic supplementary material. Sea ice extent data are publicly available from the National Snow and Ice Data Center (NSIDC)^32^ at 10.7265/N5736NV7. Mixed effects logistic regression models were run in R using code from the lme4 package^34^.

## Supplementary information

Supplementary Materials
