# Supplementary material for: Viral emergence in marine mammals in the North Pacific may be linked to Arctic sea ice reduction
Source: Sci Rep. 2019 Nov 7;9:15569. doi: 10.1038/s41598-019-51699-4 (PMC6838065; doi:10.1038/s41598-019-51699-4)
Supplement: Supplementary file 1 — Supplementary Materials [file 41598_2019_51699_MOESM1_ESM.pdf]

## Viral emergence in marine mammals in the North Pacific may be linked to Arctic sea ice reduction

E. VanWormer,[1,2] J.A.K. Mazet,[1] A. Hall, [3] V.A. Gill,[4,5] P.L. Boveng,[6] J.M. London,[6] T. Gelatt,[6] B.S. Fadely,[6] M.E. Lander,[6] J. Sterling,[6] V.N. Burkanov,[6] R.R. Ream, [6] P.M. Brock,[7] L.D. Rea,[8,9] B.R. Smith,[1] A. Jeffers,[10] M. Henstock[11], M.J. Rehberg[8], K.A. Burek-Huntington[12], S.L. Cosby[10], J.A. Hammond[11], and T. Goldstein[1,*]

## Electronic Supplementary Materials

Supplementary Table 1. Summary of marine mammals sampled and tested for phocine distemper virus (PDV) exposure (antibodies by micro neutralisation assay) and infection (viral RNA detected by PCR analyses) in the Northern Pacific Ocean and Bering, Chukchi, and Beaufort Seas from 2001 through 2016 by species, collection status (live capture, subsistence harvest, or found dead), year, and location of sampling.

| **Species [Total number sampled]** | **Number tested (serology)** | | **Years and Locations (serology)** | **Number tested (PCR)** | | **Years and Locations (PCR)** |
|---|---|---|---|---|---|---|
| | **Live capture or Subsistence harvest** | **Found Dead** | | **Live capture or Subsistence harvest** | **Found Dead** | |
| Steller sea lions (*Eumetopias jubatus*) [1,247] | 670 | - | 2001-2011, 2013<br><br>Russia, Aleutian Islands, Gulf of Alaska, Southeast Alaska | 671 | 26 | 2001- 2002, 2004-2008, 2010-2015<br><br>Russia, Aleutian Islands, Gulf of Alaska, Southeast Alaska |
| | | | | | | |
| Northern fur seals (*Callorhinus ursinus*) [886] | 281 | - | 2009-2012<br><br>Pribilof Islands (Bering Sea) | 791 | - | 2009 - 2012, 2015<br><br>Pribilof Islands (Bering Sea) |
| | | | | | | |
| Northern sea otters *(Enhydra lutris kenyoni*) [342] | 165 | 14 | 2004-2009, 2011<br><br>Aleutian Islands, Gulf of Alaska, Southeast Alaska | 168 | 135 | 2002, 2004-2012<br><br>Aleutian Islands, Gulf of Alaska, Southeast Alaska |
| | | | | | | |
| **Ice-associated seals** | | | | | | |
| Bearded seals (*Erignathus barbatus*) [51] | 3 | - | 2009<br>Chukchi Sea | 51 | - | 2009-2012, 2014<br><br>Bering and Chukchi Seas |
| Ringed seals (*Phoca hispida*) [8] | 1 | - | 2009<br>Bering Sea | 6 | 2 | 2009, 2011-2012<br><br>Bering, Beaufort, and Chukchi Seas |
| Ribbon seals (*Histriophoca fasciata*) [85] | 52 | - | 2008-2010<br>Bering Sea | 84 | - | 2008-2011, 2014, 2016<br><br>Bering and Chukchi Seas |
| Spotted seals (*Phoca largha*) [76] | 41 | - | 2009-2010<br>Bering Sea | 59 | 1 | 2009-2011, 2014, 2016<br>Bering and Chukchi Seas |

Supplementary Table 2. Sample size, seroprevalence, and viral infection prevalence estimates for Steller sea lions and all marine mammal species combined for the study period (2001-2016). Data presented in this table correspond to the prevalence estimates and error bars shown in Figure 2.

| | PDV serology results for Steller Sea Lions | | PDV serology results for all species combined | | PDV PCR results for all species combined (PDV nucleic acid detected from nasal swabs) | |
|---|---|---|---|---|---|---|
| Year | # positive (# tested) | Seroprevalence (95% exact CI) [Corresponds to black line in Figure 2a] | # positive (# tested) | Seroprevalence (95% exact CI) [Corresponds to black line in Figure 2b] | # positive (# tested)[a] | Infection prevalence (95% exact CI) [Corresponds to green line in Figure 2b] |
| 2001 | 11 (45) | 0.24 (0.13 – 0.40) | 11 (59) | 0.19 (0.10 – 0.31) | 0 (19) | 0.00 (0.0 – 0.18) |
| 2002 | 11 (40) | 0.28 (0.15 – 0.44) | 11 (40) | 0.28 (0.15 – 0.44) | na | na |
| 2003 | 38 (95) | 0.40 (0.30 – 0.51) | 38 (95) | 0.40 (0.30 – 0.51) | na | na |
| 2004 | 16 (48) | 0.33 (0.20 – 0.48) | 20 (115) | 0.17 (0.11 – 0.26) | 25 (208) | 0.12 (0.08 – 0.17) |
| 2005 | 11 (104) | 0.11 (0.05 – 0.18) | 15 (144) | 0.10 (0.06 – 0.17) | 3 (60) | 0.05 (0.01 – 0.14) |
| 2006 | 4 (8) | 0.50 (0.16 – 0.84) | 4 (8) | 0.50 (0.16 – 0.84) | 0 (10) | 0.00 (0.0 – 0.31) |
| 2007 | 3 (56) | 0.05 (0.01 – 0.15) | 8 (91) | 0.09 (0.04 – 0.17) | 0 (54) | 0.00 (0.0 – 0.07) |
| 2008 | 3 (86) | 0.04 (0.01 – 0.10) | 4 (87) | 0.05 (0.01 – 0.11) | 0 (1) | 0.00[b] |
| 2009 | 18 (53) | 0.34 (0.22 – 0.48) | 66 (161) | 0.41 (0.33 – 0.49) | 17 (122) | 0.14 (0.08 – 0.21) |
| 2010 | 7 (19) | 0.37 (0.16 – 0.62) | 35 (147) | 0.24 (0.17 – 0.32) | 13 (384) | 0.03 (0.02 – 0.06) |
| 2011 | 18 (62) | 0.29 (0.18 – 0.42) | 47 (167) | 0.28 (0.22 – 0.36) | 1 (323) | 0.003 (0.00 – 0.02) |
| 2012 | na | na | 14 (45) | 0.31 (0.18 – 0.47) | 0 (308) | 0.00 (0.00 – 0.01) |
| 2013 | 26 (54) | 0.48 (0.34 – 0.62) | 26 (54) | 0.48 (0.34 – 0.62) | 0 (54) | 0.00 (0.00 – 0.07) |
| 2014 | na | na | na | na | 0 (48) | 0.00 (0.00 – 0.07) |
| 2015 | na | na | na | na | 0 (212) | 0.00 (0.00 – 0.02) |
| 2016 | na | na | na | na | 0 (23) | 0.00 (0.00 – 0.15) |

[a] No animals PCR positive for PDV (infected live-captured or subsistence-harvested animals) were detected in nine of the 14 years with PCR samples available. A viral infection prevalence of zero in a given year could reflect the lack of PDV transmission (true zero prevalence) or could be due to an insufficient sample size to detect positive animals. Prior to our study, the only molecularly-confirmed infection with phocine distemper virus was reported in Northern sea otters off Alaska with 10% viral infection prevalence (PDV nucleic acid detected by PCR; Goldstein et al., 2009). From a large population, a sample of 139 animals in a given year would be necessary to detect a viral infection prevalence of 10% with 95% confidence and a precision of 5%. Of the nine years with zero infection prevalence, the sample size was larger than this target in two years (2012 and 2015). In three of the remaining seven years with zero prevalence (2007, 2013, and 2014), the sample size exceeded 35 animals, the number necessary for 95% confidence and a precision of 10%.

[b] A 95% confidence interval was not calculated for this year as only one animal was tested.

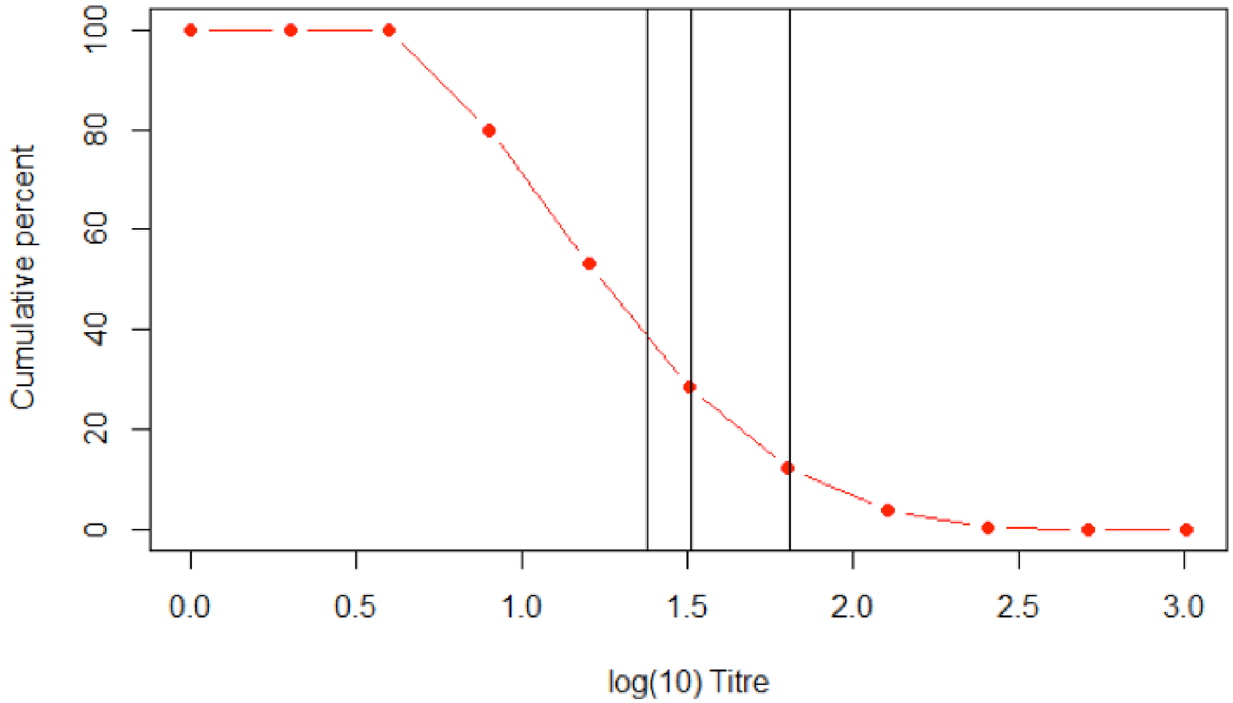


Supplementary figure 1. Cumulative distribution of log10-transformed Phocine distemper virus (PDV) serologic titres for marine mammals sampled in the North Pacific Ocean 2001-2013. Vertical lines represent titres of 1:16 (log(10) titre = 1.38), 1:32 (log(10) titre = 1.51) and 1:64 (log(10) titre = 1.81) respectively.

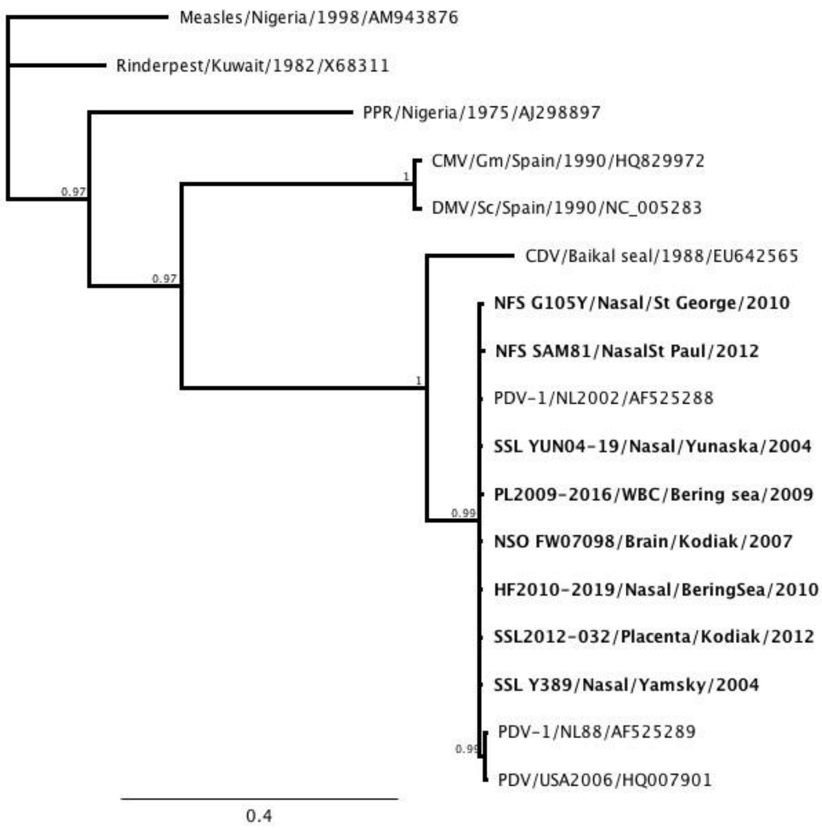


Supplementary figure 2. Phylogenetic tree showing the relationship of a 389bp fragment of the phosphoprotein gene of the Phocine distemper sequences obtained from representative samples from all species, regions and years (in bold) from the North Pacific, compared to other known Phocine distemper and morbilli viruses from the Genbank database. Corresponding bootstrap values are shown at the bases of each branch based on 1000 re-samplings of the data. The scale bar indicates the number of nucleotide substitutions per site; measles (Genbank accession no. AM943876) was designated as the outgroup. Genbank accession numbers for sequences obtained in this study are provided in the Data, Code and Materials section.

a)

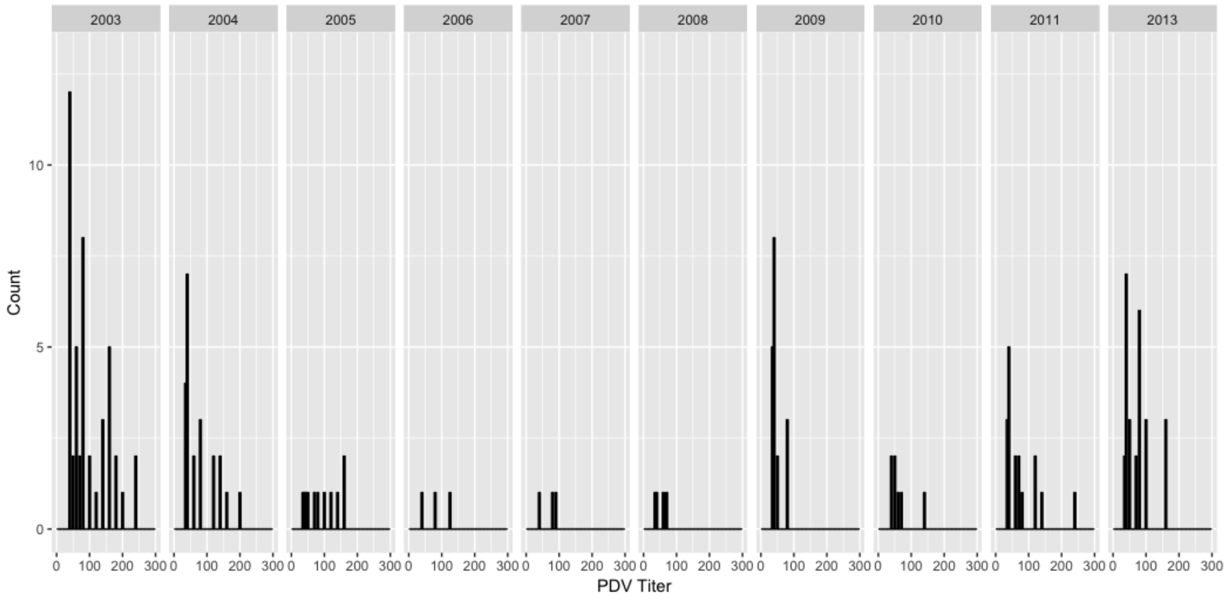
2003
2004
2005
2006
2007
2008
2009
2010
2011
2013
Count
PDV Titer

b)

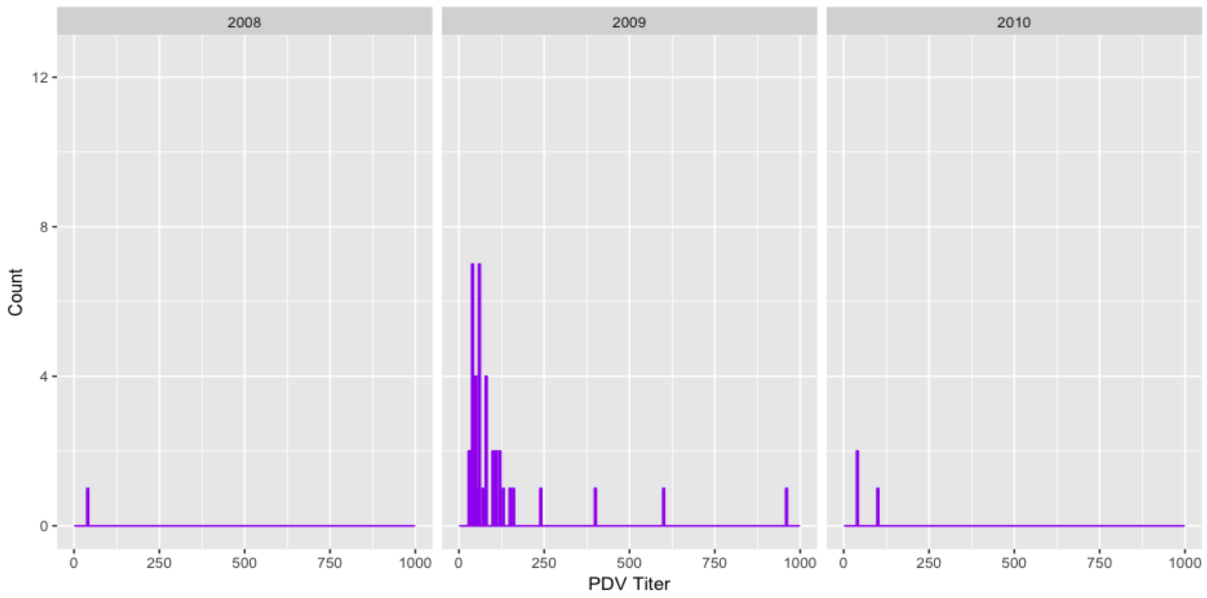
2008
2009
2010
Count
PDV Titer

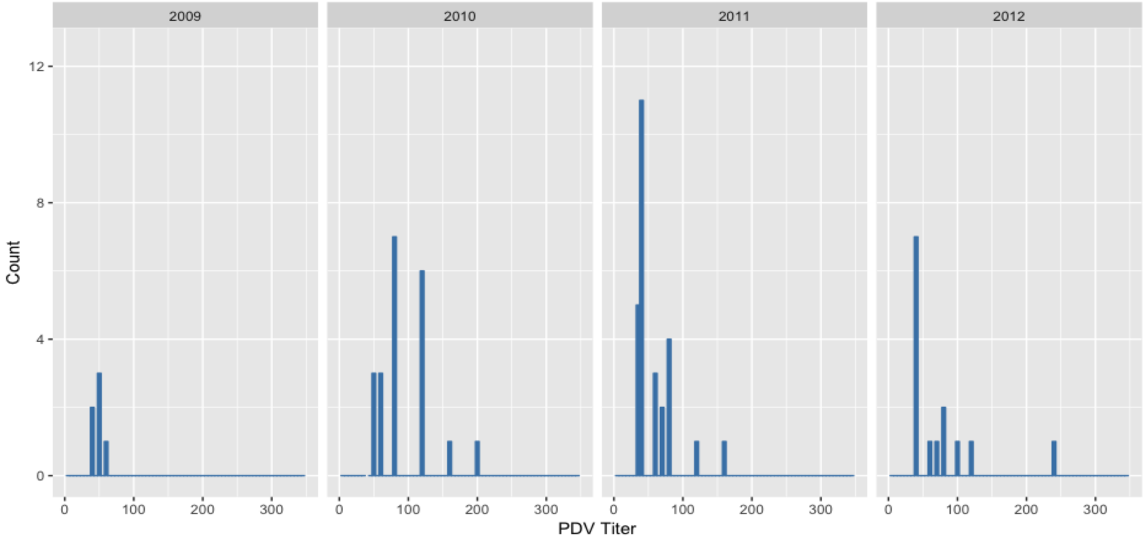


**c)**

Supplementary figure 3. Histograms of PDV serologic titres by year in seropositive a) Steller sea lions, b) ice-associated seals, and c) northern fur seals sampled in the Northern Pacific Ocean 2003-2013. Animals with a serologic titre ≥ 1:32 were considered to be seropositive.
